# Supplementary material for: Large Room Temperature Anomalous Nernst Effect Coupled with Topological Nernst Effect from Incommensurate Spin Structure in a Kagome Antiferromagnet
Source: Adv Sci (Weinh). 2026 Jan 12;13(17):e22151. doi: 10.1002/advs.202522151 (PMC13042588; doi:10.1002/advs.202522151)
Supplement: Supplementary file 1 — Supporting File: advs73792‐sup‐0001‐SuppMat.pdf. [file ADVS-13-e22151-s001.pdf]

SUPPLEMENTARY INFORMATION

Large room temperature anomalous Nernst effect coupled with topological Nernst effect from incommensurate spin structure in a kagome antiferromagnet

Jiajun Ma[1], Jiaxing Liao[1], Yazhou Li[1], Yuwei Zhang[1], Jialu Wang[1], Jinke Bao[1], Yan Sun[2,#], Shuang Jia[3,*], Yuke Li[1,*]

1. *School of Physics and Hangzhou Key Laboratory of Quantum Matters, Hangzhou Normal University, Hangzhou 311121, China*
2. *Shenyang National Laboratory for Materials Science, Institute of Metal Research, Chinese Academy of Sciences, Shenyang, 110016, China*
3. *International Center for Quantum Materials, School of Physics, Peking University, Beijing 100871, China*

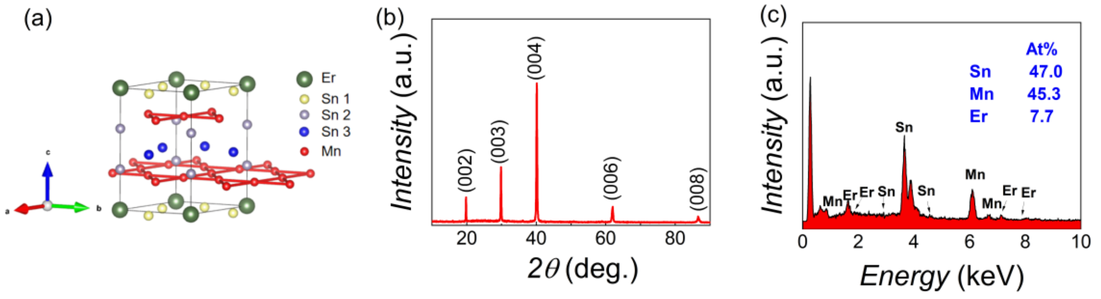

Figure S1 (a) The sketch picture of crystal structure of $ErMn_6Sn_6$. (b) The (00l) diffraction peaks of single crystal $ErMn_6Sn_6$. (c) The energy-dispersive X-ray (EDX) spectroscopy of $ErMn_6Sn_6$ sample and its actual component.

$ErMn_6Sn_6$(EMS) crystallizes in a hexagonal structure with space group P6/mmm, featuring Er and Sn1 atoms on the same layer. Along the c-axis, the crystal structure features an alternating Mn-Sn2-Sn3-Sn2-Mn atomic layers, forming two distinct kagome Mn layers, while the Sn2 and Sn3 atoms adopt a hexagonal structure, as seen in Figure S1(a). The single crystal diffraction patterns clearly show a set of (00l) peaks without any extra impurity phase in Figure S1(b). The energy-dispersive X-ray (EDX) spectroscopy confirms that the actual ratio of Er, Mn and Sn is close to 1:6:6 in Figure S1(c).

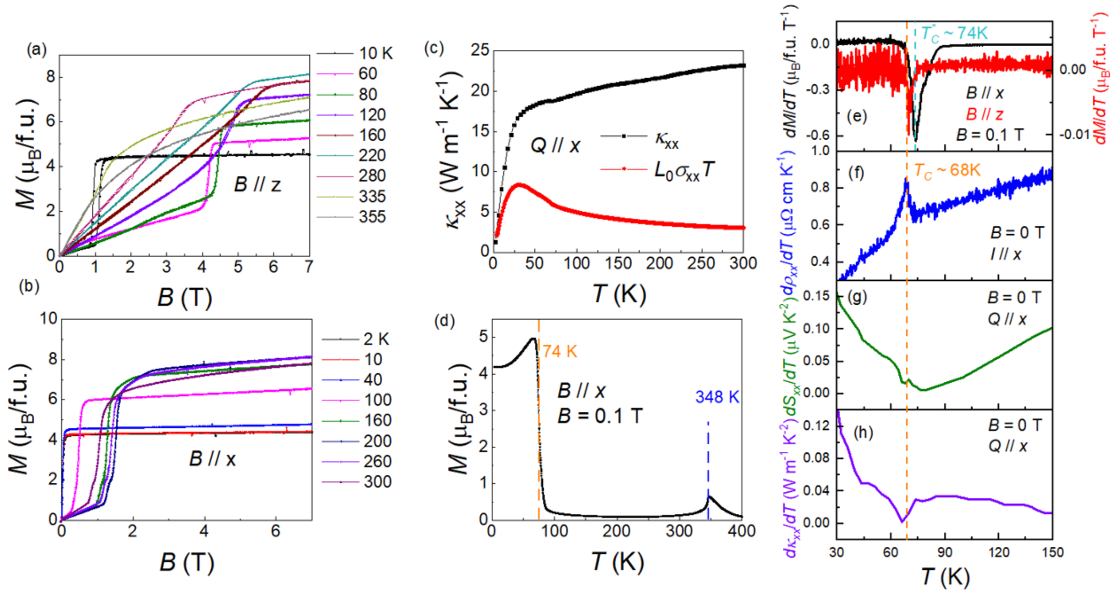

Figure S2 (a) and (b) the magnetization vs. the applied magnetic fields with both B // z-axis and B // x-axis, respectively, over a temperature range from 2 to 300 K. (c) The thermal conductivity and the electronic thermal conductivity as functions of temperature as heat flows the x-axis. (d)Temperature dependence of magnetization as *B* // *x* -axis, at 0.1 T. (e) The differentiation of magnetization as functions of temperature as B // z-axis (Right) and B // x-axis (left). (f)-(h) For heat/current along x-axis, the differentiation of resistivity (f), thermopower (g) and thermal conductivity (h) as function of temperature at zero field.

EMS undergoes a field-induced ferrimagnetic phase transition at low temperatures when B // z-axis. With the increase of magnetic fields, the magnetization starts to linearly increase, followed by a sudden jump associated with the ferrimagnetic transition, and finally tends to saturate at higher

magnetic fields. Above 100 K, the system transitions from an AFM to a FIM state[1, 2] in Figure S2(a). A similar evolution is observed for B // x-axis, though saturation occurs at lower field values within the ab-plane (Figure S2(b)). The weak magnetic phase transitions observed in the low-field magnetization curve are attributed to the formation of a triplet-spiral order[3]. It is noteworthy that neutron diffraction studies have confirmed the presence of a dominant spiral AFM state in EMS, characterized by an incommensurate spin texture.

The thermal conductivity exhibits an overall linear temperature dependence with a subtle anomaly near the Curie temperature T_C, followed by a sharp drop below 30 K. The total thermal conductivity is composed of electrons, phonons, and magnon, expressed as $\kappa=\kappa_e+\kappa_p+\kappa_m$. Using the Wiedemann-Franz law, the electronic thermal conductivity can be estimated from the electrical conductivity[4]. As shown in Figure S2(c), the longitudinal thermal conductivity significantly exceeds the electronic thermal conductivity at high temperatures, but the two values gradually approach each other at low temperatures. This behavior suggests that phonons and magnons thermal transport dominates in high temperatures regime, while electronic thermal conductivity becomes predominant at lower temperatures. Magnetization curves M(T) with zero-field-cooled mode are performed at a magnetic field of 0.1 T along the x-axis, in Figure S2(d). The dip on the temperature derivative of out-plane magnetization and in-plane magnetization marks $T_C \sim 68$ K and $T_C \sim 74$K, respectively, as seen in Figure S2(e). These results confirm the ab-plane as the easy magnetization plane in the ferrimagnetic state. The differentiation of resistivity, thermopower and thermal conductivity also observes a kink at 68 K, as seen in Figure S2(f-h).

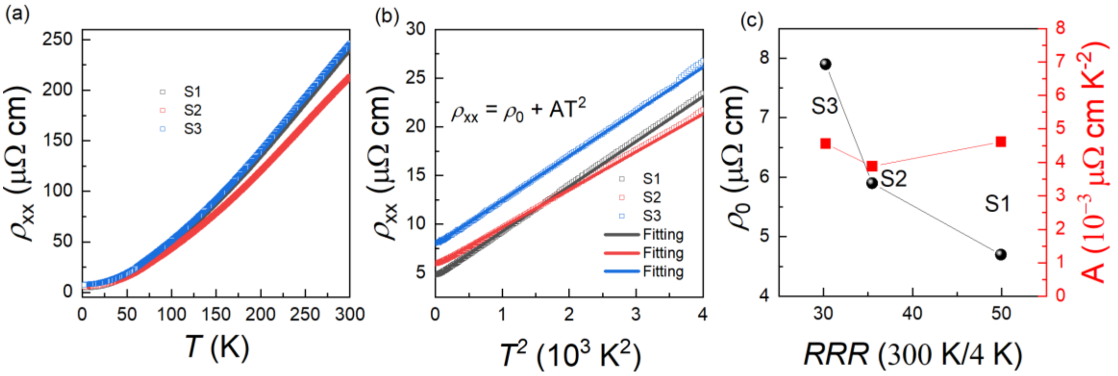

Figure S3 (a) Temperature-dependent resistivity of three EMS samples (S1-S3). (b) The low-T resistivity as a function of quadratic temperature. (c) Residual resistivity (ρ_0) and parameter A of three samples as a function of the residual resistivity ratio (RRR).

The temperature-dependent resistivity of all three samples exhibits metallic behavior, as illustrated in Figure S3(a). In low temperature regime, the resistivity follows a quadratic temperature dependence, $\rho = \rho_0 + AT^2$, as shown in Figure S3(b). Here, ρ_0 represents the residual resistivity influenced by sample defects, while the coefficient A is an intrinsic parameter related to the electronic structure near the Fermi level. Although significant variations in ρ_0 and the residual resistivity ratio (RRR) among the samples reflect differences in crystalline quality (Fig. S3(c)), the A values remain nearly identical. Furthermore, the carrier densities of the three samples are comparable, as evidenced in Figure S4(e), indicating that the Fermi energy is consistently positioned across different samples.

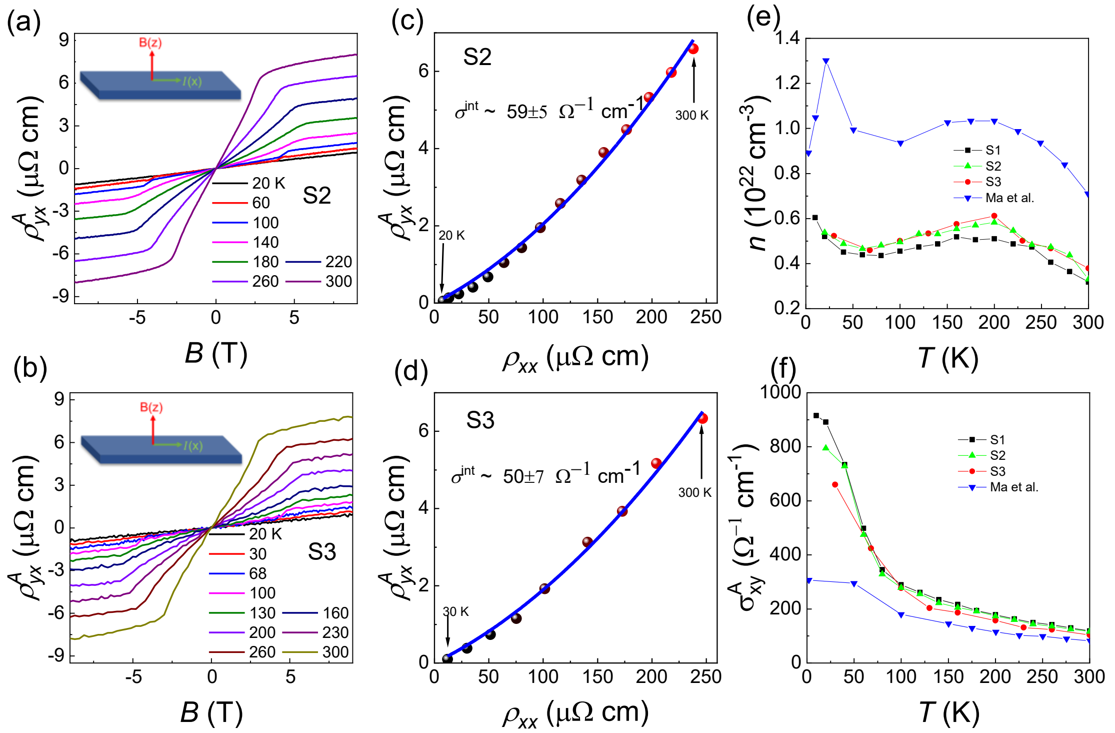

Figure S4 Hall resistivity of S2 (a) and S3 (b) as function of magnetic field (B // z-axis) at temperature form 20 K to 300 K. (c)(d) The anomalous Hall resistivity ρ_{yx}^{A} plotted against the longitudinal resistivity ρ_{xx} for S2 (from 20 K) and S3 (from 30 K) to 300 K. The bule line represents the polynomial fitting of the data by Eq (S2), which gives the intrinsic anomalous Hall conductivity σ^{int} = 59±5 Ω^{-1} cm^{-1} for S2 and 50±7 Ω^{-1} cm^{-1} for S3. The temperature-dependent carrier concentrations(e) and anomalous Hall conductivity(f) for the three samples, compared with Ma et.al reported values[5].

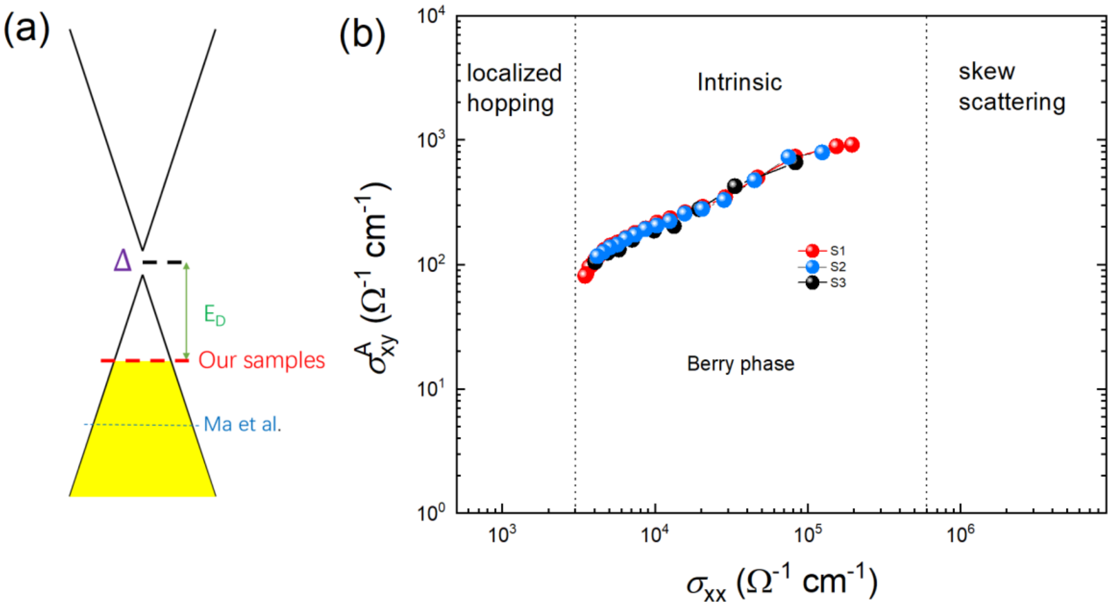

Figure S5 (a)The Fermi levels of our samples and ref [5] deviate from the Dirac cone. Dirac cones are gapped out in the presence of SOC and Ed represents the distance from the center of the energy gap to the Fermi energy. (b) Scaling of the AHC with longitudinal conductivity for the three EMS samples. The three regions are distinguished

by longitudinal conductivity as follows: the low conductivity region is the dirty metal region where localized hopping plays a role; the intermediate region is the good metal region where the intrinsic mechanism dominates; and the high conductivity region is where skew scattering predominates.

The anomalous Hall resistivity (AHR) of samples S2 and S3, measured under an out-of-plane magnetic field (B // z), exhibits behavior fully consistent with sample S1, as shown in Figure S4(a) and (b). The AHR were much larger than the prior studies (Ma et. al.)[5], and were analyzed using the relation: $\rho_{yx}^{A} = \sigma_{xy}^{int}\rho_{xx}^{2} + \beta^{skew}\rho_{xx}$, where σ^{int} is the intrinsic anomalous Hall conductivity (AHC), and β^{skew} denotes the skew scattering contribution parameter. By fitting this expression across the temperature range of 30 K to 300 K, the intrinsic AHC values for S2 and S3 are determined to be approximately 50-60 $\Omega^{-1}\,cm^{-1}$, closely matching each other as shown in Figure S4(c) and S4(d). Moreover, the carrier concentrations of all three samples are found to be almost consistent. Notably, however, the carrier concentration reported in prior studies (Ma et. al.) [5] is nearly an order of magnitude higher than that observed in our samples, as illustrated in Figure S4(e).

Figure S5(a) demonstrates that the intrinsic Hall conductivity in our sample is significantly larger than values reported in previous works[5]. In EMS, the dominant hole-like carriers, which is evidenced by the positive signs of both the thermopower and the Hall coefficient, imply that the Fermi level lies below the Chern gap. Introducing electron doping[6] can shift the Fermi level closed upward toward the Chen gap, thereby enhancing the Berry curvature and leading to a pronounced increase in the intrinsic Hall conductivity, as illustrated in Figure S5(a). The doping effect, potentially originating from differing sample preparation methods, thus highlights practical strategy for optimizing Berry curvature and anomalous Hall response through controlled carrier concentration tuning.

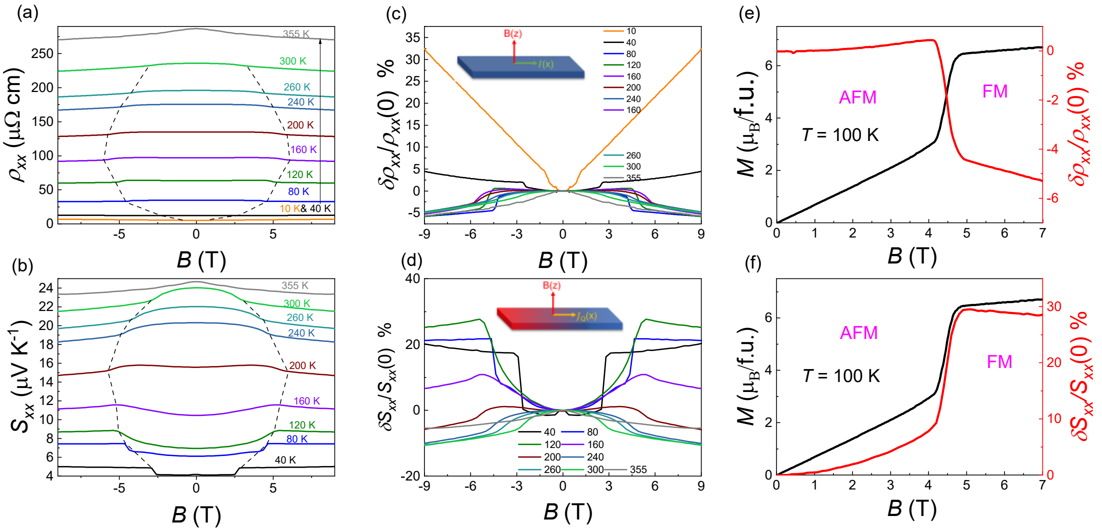

Figure S6 Longitudinal resistivity ρ_{xx}(a) and thermopower S_{xx}(b) as a function of magnetic field at different temperatures. (c) Magneto resistivity ratio $\frac{\delta\rho_{xx}}{\rho_{xx}(0)} = \frac{\rho_{xx}(H)-\rho_{xx}(0)}{\rho_{xx}(0)}$ and (d)magneto thermopower ratio $\frac{\delta S_{xx}}{S_{xx}(0)} = \frac{S_{xx}(H)-S_{xx}(0)}{S_{xx}(0)}$(d) as a function of magnetic field at different temperatures. (e) The moment (left-axis) and magneto resistivity ratio (right-axis) and (f) the moment(left-axis) and magnetothermopower ratio(right-axis) as a function of magnetic field at 100 K.

Figure S6(a) and (b) display the magnetic field dependence of the resistivity ρ_{xx} and thermopower S_{xx} in EMS at different temperatures. Both quantities exhibit a pronounced jump at a critical magnetic field, corresponding to the transition from antiferromagnetic (AFM) to ferrimagnetic (FiM) phase. This behavior suggests a significant modification in carrier scattering, likely originating from spin-flip processes. As shown in Figure S6(c) and (d), the magnetoresistance $\frac{\delta\rho_{xx}}{\rho_{xx}(0)}$ reaches approximately 32% at 10 K and 9 T, while the magneto-thermopower $\frac{\delta S_{xx}}{S_{xx}(0)}$, attains 25% at 120K and 9 T. These values are comparable to those reported in YMn_6Sn_6[7] and $ScMn_6Sn_6$[8]. At T = 100 K, as the system transitions into the FiM state (Figure S6(e) and (f)), the magneto-resistivity starts to decrease, while the magneto thermopower exhibits the opposite trend, similar to the observation in $MnBi_2Te_4$[9]. This behavior can be explained by magnon-mediated effects: magnons transfer momentum to charge carriers, thereby enhancing the thermopower S. The magnitude of this enhancement scales with τ_m/τ_{em}, where τ_m is the magnon relaxation time and τ_{em} is the electron-magnon scattering relaxation time. During the polarization process from the AFM to the FiM state, magnon scattering is suppressed, leading to an increase in τ_m and a enhancement in S.

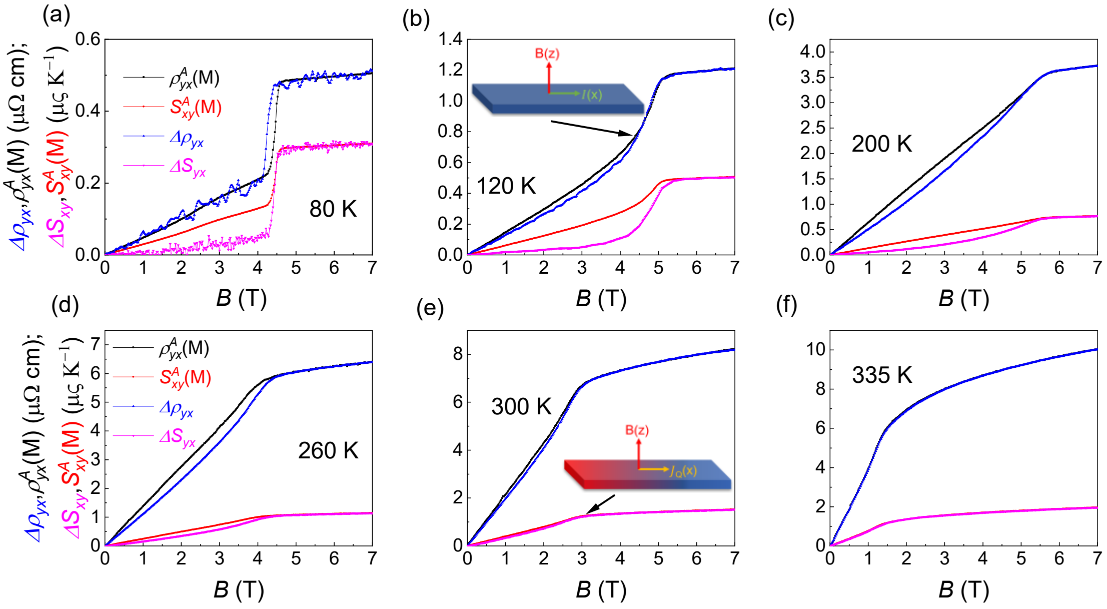

Figure S7 (a-f) The isolated topological Hall and topological Nernst effects from 80 to 355 K. $\Delta\rho_{yx}$ (blue line)represents the combined contribution of topological Hall resistivity and the anomalous Hall resistivity; $\rho^A_{yx}(M)$ (black line) represents the anomalous Hall resistivity component that is proportional to the magnetization; Similarly, ΔS_{xy}(pink line) represents the total signal comprising both topological Nernst and anomalous Nernst effects; while $S^A_{xy}(M)$ (red line) corresponds to the anomalous Nernst signal.

The topological Hall effect and topological Nernst effect can be obtained by subtracting the normal component and the anomalous component related to magnetization from the total effect, as illustrated in Figure S7. Generally, only the normal term and the anomalous term exist at high magnetic fields, allowing us to extract the topological term. As the temperature increases, the difference between the $\Delta\rho_{yx}$ (or ΔS_{xy}) and the ρ^A_{yx}(M) (or S^A_{xy}(M)) becomes increasingly significant, gradually diminishing near T_N.

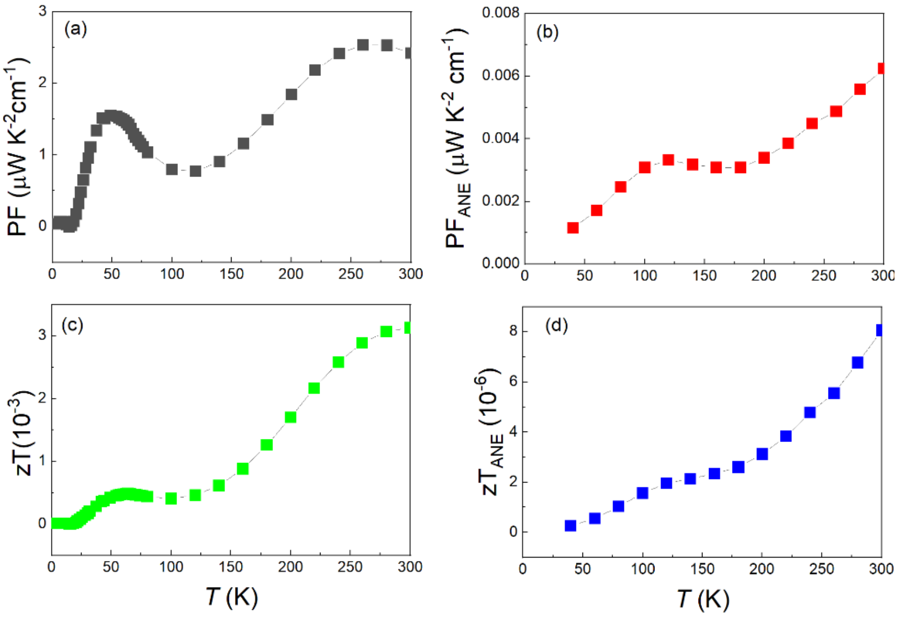

Figure S8 The temperature dependence of longitudinal performance factor (a), anomalous transverse performance factor (b), longitudinal zT (c) and transverse zT_{ANE} (d) values for EMS.

In Figure S8, the EMS exhibits relatively modest performance parameters. Over the temperature range of 4–300 K, its longitudinal power factor (PF) and zT are approximately comparable to the values observed in kagome magnets $Co_3Sn_2S_2$ (PF~5μW/K^2 cm; zT~10^{-3} at 80 K)[10] and $TbMn_6Sn_6$ (PF~3μW/K^2 cm; zT~10^{-2} at 300 K)[11]. Meanwhile, Its transverse PF_{ANE} and zT_{ANE} are one orders of magnitude lower than $Co_3Sn_2S_2$ (PF_{ANE}~0.05 μW/K^2 cm; zT_{ANE}~10^{-5} at 80 K) and $TbMn_6Sn_6$(zT_{ANE}~10^{-5} at 300 K).

References:

[1] K. Fruhling, A. Streeter, S. Mardanya, et al., "Topological Hall effect induced by chiral fluctuations in $ErMn_6Sn_6$," *Physical Review Materials, 8 (2024) 094411*.

[2] S.X.M. Riberolles, T. Han, T.J. Slade, et al., "New insight into tuning magnetic phases of RMn_6Sn_6 kagome metals," *npj Quantum Materials*, 9 (2024) 42.

[3] S.S. Samatham, J. Casey, A.M. Szucs, et al., "Perturbation-tuned triple spiral metamagnetism and tricritical point in kagome metal $ErMn_6Sn_6$," *Communications Materials*, 5 (2024) 113.

[4] A. Jaoui, B. Fauqué, C.W. Rischau, et al., "Departure from the Wiedemann–Franz law in WP_2 driven by mismatch in T-square resistivity prefactors," *npj Quantum Materials*, 3 (2018) 64.

[5] W. Ma, X. Xu, J.-X. Yin, et al., "Rare Earth Engineering inRMn_6Sn_6(R=Gd−Tm, Lu) Topological Kagome Magnets," *Physical Review Letters*, 126 (2021).

[6] J.-X. Yin, W. Ma, T.A. Cochran, et al., "Quantum-limit Chern topological magnetism in $TbMn_6Sn_6$," *Nature*, 583 (2020) 533-536.

[7] S. Roychowdhury, A.M. Ochs, S.N. Guin, et al., "Large room temperature anomalous transverse thermoelectric effect in kagome antiferromagnet YMn_6Sn_6," *Advanced Materials*, 34 (2022) 2201350.

[8] R.P. Madhogaria, S. Mozaffari, H. Zhang, et al., "Topological Nernst and topological thermal Hall effect in rare-earth kagome $ScMn_6Sn_6$," *Physical Review B*, 108 (2023) 125114.

[9] H. Zhang, C. Xu, S. Lee, Z. Mao, X. Ke, "Thermal and thermoelectric properties of an antiferromagnetic topological insulator $MnBi_2Te_4$," *Physical Review B*, 105 (2022) 184411.

[10] S.N. Guin, P. Vir, Y. Zhang, N. Kumar, et al., "Zero-Field Nernst Effect in a Ferromagnetic Kagome-Lattice Weyl-Semimetal $Co_3Sn_2S_2$," *Advanced Materials*, 31 (2019) 1806622.

[11] X. Xu, J.-X. Yin, W. Ma, et al., "Topological charge-entropy scaling in kagome Chern magnet $TbMn_6Sn_6$," *Nature communications*, 13 (2022) 1197.
